# Silver Nanowires from Sonication-Induced Scission

**DOI:** 10.3390/mi10010029

**Published:** 2019-01-04

**Authors:** Yuehui Wang, Xing Yang, Dexi Du

**Affiliations:** 1 School of Materials and Energy, University of Electronic Science and Technology of China, Chengdu 610054, China; shirleywyh@126.com (X.Y.); julydlyx@126.com (D.D.); 2 Department of Materials and Food, University of Electronic Science and Technology of China Zhongshan Institute, Zhongshan 528402, China

**Keywords:** silver nanowires, sonication-induced scission, ultrasound power, fragmentation

## Abstract

Silver nanowires (AgNWs) have great potential to be used in the flexible electronics industry for their applications in flexible, transparent conductors due to high conductivity and light reflectivity. Those applications always involve size which strongly affects the optical and electrical properties of AgNWs. AgNWs of mean diameter 70 nm and mean length 12.5 μm were achieved by the polyol solvothermal method. Sonication-induced scission was used to obtain the small size AgNWs. The relationship between the size of AgNWs and the ultrasonic time, ultrasonic power, and concentration of AgNWs were studied. The results show that the length of AgNWs gradually reduces with the increase of the ultrasonic time and ultrasonic power, and with the decrease of concentration of AgNWs. Meanwhile, there is an existence of a limiting length below which fragmentation of AgNWs no longer occurs. Further, the mechanics of sonication-induced scission for the fragmentation of AgNWs was discussed.

## 1. Introduction

With the development of portable electronic devices, indium tin oxide (ITO) conductive films have gradually failed to meet people’s needs due to their brittleness and flexibility [1,2]. Therefore, many conductive materials that are expected to replace ITO have been studied, such as graphene [3,4,5], silver nanowires (AgNWs) [6], carbon nanotubes [7], and metal grids [8,9,10]. Tiefenauer et al. fabricated stretchable plasmonic-coupled graphene sensors which are based on gold nanowire arrays on a polydimethylsiloxane platform with an added graphene monolayer on top. This plasmonic sensor exhibited ultrahigh sensitivity to applied strain, which can be detected by shifts in the plasmonic-enhanced Raman spectrum and enabled the detection of adsorbed molecules on nonplanar surfaces through graphene-assisted surface enhanced Raman spectroscopy [3]. Meanwhile, there are also some approaches to make stiff materials into soft materials via irradiation [11], electron-beam-assisted [12], etc.

In recent years, people have become more and more inclined to use a conductive network composed of nanowires. Considering various issues, AgNWs have received extensive attention [6,7,8,9,13,14,15,16,17,18,19,20,21,22,23,24,25]. Due to the high conductivity and flexibility of AgNW networks, AgNWs have shown promise in high visual transparency and conductivity essential for optoelectronic applications, such as solar cells, radio frequency identification technology, organic light-emitting devices and so on [7,8,9,13,14,15,16,17]. Maish et al. fabricated semitransparent organic solar cells with AgNW percolation networks as bottom and top electrodes by an inkjet-printed process and demonstrated that the power conversion efficiency can be 4.3% for 1 cm^2^ area [6]. Huang et al. reported gravure printing of water-based AgNW ink on a flexible substrate [13]. The printed AgNW patterns on the flexible substrate showed excellent flexibility under repeated bending [13].

As we all know, the length of silver nanostructures affects strongly their properties and applications [7,8,9,13,14,15,16,17]. It has been reported in many literatures that the photoelectric properties of AgNW transparent conductive films improve with an increase in length of AgNWs [13,14,15,16,17]. A number of methods have been developed for the preparation of AgNWs having a controllable structure such as liquid phase polyol methods, hydrothermal methods, templating methods and so on [17,18,19,20,21,22,23,24,25]. The preparation of AgNWs with different sizes can be achieved by adjusting the concentration, amount, reaction temperature and time of the reactants, and reducing agents, agents and surfactants [17,18,19,20,21,22,23,24,25]. However, the controllable preparation of silver nanostructures is still changing, because most of the prepared AgNWs have a large size distribution range [23,24,25]. 

Sonication is widely applied to disperse materials in liquid media due to the ultrahigh shear rate attained during cavitation events [26,27,28,29,30,31,32]. However, sonication can also induce the scission of the materials that are imploding cavitation bubbles. Sonication-induced scission is often used to solve the problem of the scission of fiber-like structures with high aspect ratio, including the exfoliation and scission of carbon nanotubes [26,27,28,29,30,31,32]. Recently, the kinetics of nanotube and microfiber sonication-induced scission and the effects of sonication-induced scission on the morphology were discussed [23,24,25,26,27,28,29,30]. Heller et al. reported that the scission process was diameter-selective by gel electrophoresis and column chromatography analysis of ultrasonic treated single-walled carbon nanotubes, that the separation by diameter is concomitant with length fractionation and that the distribution of nanotubes changes with the ultrasonic treatment time [30]. Hennrich et al. investigated the effect of ultrasound on the fracture of nanotubes by studying the length-selective aqueous suspension of single-walled carbon nanotubes, and it was found that the cavitation-related strain forces are proportional to the square of the length of the nanotubes [31]. When the strain force is lower than the critical value that can destroy the nanotubes, the fracture stops and the maximum tube length distribution initially exhibits a power-law dependence on the ultrasound time (*l*_M_ ≈ *t*^−0.5^) [29]. Stegen proposed a length-dependent scission rate theory by taking the movement of the carbon nanotubes into account, and it is pointed out that the fracture rate depends on the length and tensile strength of the carbon nanotubes [32]. To the best of our knowledge, there have been few reports of AgNWs specifically describing AgNWs from sonication-induced scission [33,34].

The research content of this paper provided an idea for obtaining AgNWs with controllable size. Sonication-induced scission of AgNWs was carried out, and the effect of ultrasonic time and power-concentration of AgNWs on the size of AgNWs were investigated.

## 2. Materials and Methods

### 2.1. Materials

Silver nitrate (≥99.8%) was purchased from Guangzhou Jinhuada Chemical Reagent Co., Ltd., Guangzhou, China. Poly(vinylprrolidone) (PVP, *M*w ≈ 10,000), was purchased from Jinan Jiage Biological Technology Co., Ltd., Guangzhou, China. Ferric chloride (≥99.5%) was purchased from Shanghai Hongshun Biological Technology Co., Ltd., Shanghai, China, and ethylene glycol (≥99.7%) and ethanol absolute (99.7%) were purchased from Jinan Liyang Chemical Co., Ltd., Jinan, China. All the chemicals were used as received. 

### 2.2. Methods

#### 2.2.1. Preparation Method of Silver Nanowires

AgNWs were prepared by the polyol solvothermal method. First, 0.0649 g FeCl_3_ was dissolved in 40 mL of EG and diluted 50 times to get solution A. Then 0.6795 g AgNO_3_ and 1.0193 g PVP were dissolved in 40 mL of ethylene glycol and solution A, respectively. The obtained mixture was then transferred into a 100 mL reaction kettle and reacted at 160 °C for 3 h until the reaction was completed. After the completion of the reaction, the sample was removed and cooled down to room temperature. AgNWs were purified by centrifuging at 6000 rpm for 20 min in the presence of acetone and ethanol three times. At last, the AgNWs were re-dispersed in deionized water.

#### 2.2.2. Sonication-Induced Scission of Silver Nanowires

For ultrasonication treatments, An AgNW aqueous solution was diluted to 0.0084–1.67 mg·mL^−1^ and sequentially underwent ultrasonication for 1–6 h at a power of 0–480 W. The ultrasonication was carried out with a bath type sonicator (JP-120ST, 0–600 W, 28/40 kHz, Shenzhen Jiemeng Cleaning Equipment Co., Ltd., Shenzhen, China). The length of individual AgNWs in the images was measured manually according to an image processing software ((ImageJ Software. version 1.46, developed by National Institutes of Health, Bethesda, MA, USA). The morphologies of AgNWs were characterized with scanning electron microscope (SEM, zeiss sigma 500, Carl Zeiss, Jena, Germany) measurement. X-ray diffraction (XRD, DIFFRRACTOMETER, Rigaku Co., Tokyo, Japan) was used to measure the phase structures.

## 3. Results and Discussion

**Figure 1:**
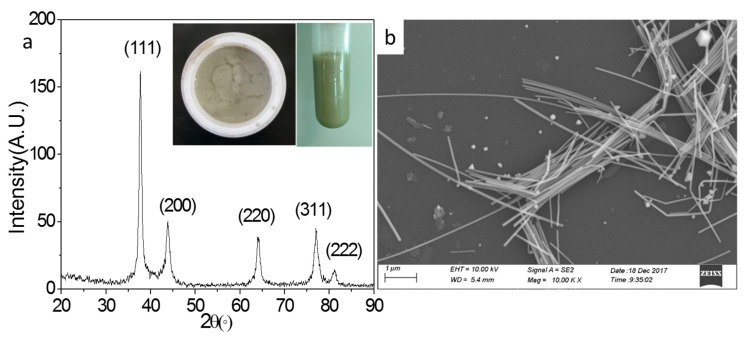
XRD (**a**) and SEM (**b**) of AgNWs. The inserted photos are the as-synthesized AgNW solution before (**left**) and after purified (**right**), respectively.

**Figure 2:**
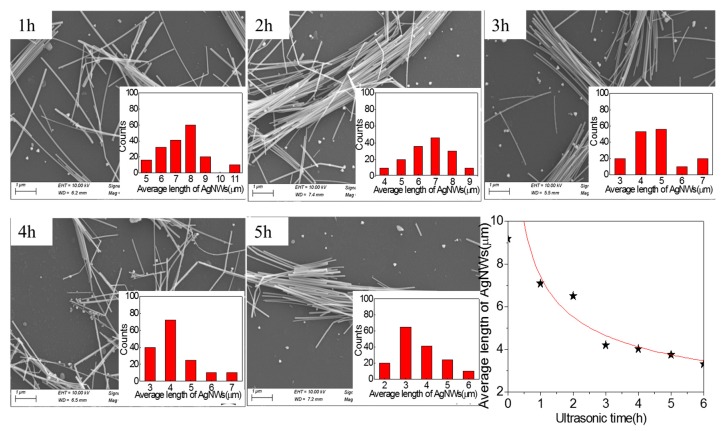
SEM images of AgNWs (1.67 mg·mL^−1^) treated with ultrasonic power 100 W and different ultrasonic time and the relationship of average length of AgNWs with ultrasonic time.

Figure 1a shows the XRD pattern of as-synthesized AgNWs. The inserted photos are the AgNW solution before (left) and after purified (right), respectively. There are five distinct diffraction peaks in the spectrum. The 2θ are 38.12°, 44.32°, 65.54°, 77.40°, and 81.56°, respectively, which is consistent with the XRD standard spectrum of silver. The corresponding diffraction planes are (111), (200), (220), (311), and (222), respectively. It is shown that the as-prepared AgNW has a face-centered cubic structure. The colors of the AgNW solution before and after purified are grey and gray-green, respectively. Figure 2b shows the SEM image of the AgNWs. AgNWs of mean diameter 70 nm and mean length 12.5 μm were achieved.

Figure 2 shows SEM images of AgNWs (1.67 mg·mL^−1^) treated with ultrasonic power of 100 W at different ultrasonic times and the relationship of average length of AgNWs with ultrasonic time. The inserted images are the length distribution of AgNWs. It has been observed that the average length of AgNWs gradually becomes shorter with the increase of the ultrasonic time, finally changing little after prolonged sonication. The average length of AgNWs reduced to 3.31 μm after sonicated for 6 h. Meanwhile, interesting phenomena are observed, the range of the length distribution of AgNWs after sonicated has been found to be significantly narrower than in the initial population of AgNWs, the long AgNWs were fragmented and shorter nanowires remained with similar lengths. It is indicated that the decrease in length is dominated by a mechanical shearing process resulting from the fluid flow, rather than a degradation process caused by thermal or chemical effects of sonication [25]. The length of AgNWs from sonication-induced scission decreases obviously within the initial three hours, after that, the length of AgNWs slightly reduced. The reason is that the short AgNWs can be dragged away easily by the fluid flow. By fitting the scatter data, we obtain a power law function between the average length of the AgNWs and the ultrasonic time, as shown in Equation (1).

(1) $$L(t)\propto t^{-0.42}$$

where *t* is the ultrasonic time and *L*(*t*) represents the average length of AgNWs at different times. Lucas and coworkers studied the scission law of ultrasonically fractured carbon nanotubes and found that the average length of carbon nanotubes scales as *t^−n^*, with *n* ≈ 0.2, which is different from the scission law of AgNWs we have derived [29]. During ultrasound, ultrasound energy (E_US_) is the product of ultrasound time (T_US_) and ultrasound power (P_US_), E_US_ = T_US_ × P_US_ [29]. Therefore, under the condition that the ultrasonic power is constant, the ultrasonic energy is proportional to the ultrasonic time. From this we conclude that the trend between the average length of the ultrasonically treated AgNWs and the ultrasonic energy is the same as the trend between the average length and the ultrasonic time.

Previous works have shown that the scission or dispersion of carbon nanotubes is dependent on sonication energy, rather than sonication time [30]. However, our experimental results show that when the length of the nanowire is less than a certain value, the scission of the nanowires with ultrasonic power of 100 W is independent of the ultrasonic time. We think that the difference may be due to the length and properties of the materials.

**Figure 3:**
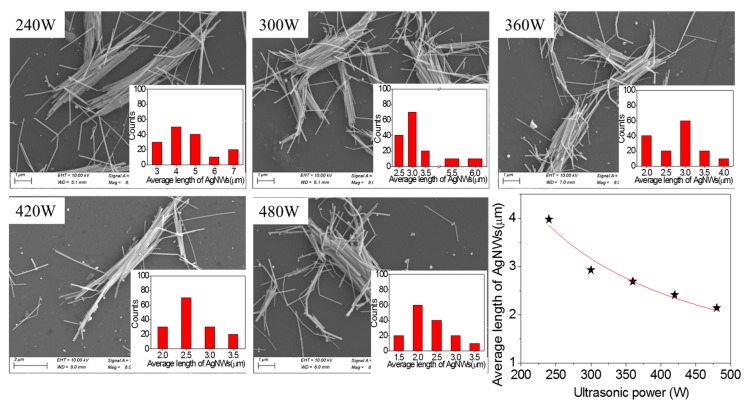
SEM images of AgNWs (1.67 mg·mL^−1^) sonicated for 1.5 h and different ultrasonic powers and the relationship of average length of AgNWs with ultrasonic power.

Figure 3 shows SEM images of AgNWs (1.67 mg·mL^−1^) sonicated for 1.5 h and different ultrasonic powers and relationship of average length of AgNWs with ultrasonic power. The inserted images are the length distribution of AgNWs. Compared with Figure 2, it is clear that the ultrasonic power has significant effect on the length of AgNWs from sonication-induced scission. When the ultrasonic power is 240 W, the length of AgNWs reduces from 11.8 μm to 3.98 μm. When the ultrasonic power is 480 W, the average length of AgNWs reduces to 2.14 μm. When the ultrasonic power of 300–480 W were applied, it was found that the length of AgNWs linearly decreased, however, the degree of the length reduction is low, which may be because the size of the nanowires was small after ultrasonic power of 300 W. The dispersion of AgNWs is not good, and some clusters of AgNWs are observed. Similarly, we performed a fit on the obtained scatter data, and obtained the power law function shown in Equation (2) with a fitness of 0.88, where *p* represents the ultrasonic power and *L*(*p*) represents the average length of the AgNWs after different ultrasonic power treatments.

(2) $$L(p)\propto p^{-0.88}$$

Table 1 shows the samples and different applied sonication energies. The sonication energy to the AgNW solution was controlled by varying the sonication powers from 240 to 480 W. According to the previous formula, we can infer that the changing trend of the average length of AgNWs and the acoustic energy is similar to that of ultrasonic power.

**Figure 4:**
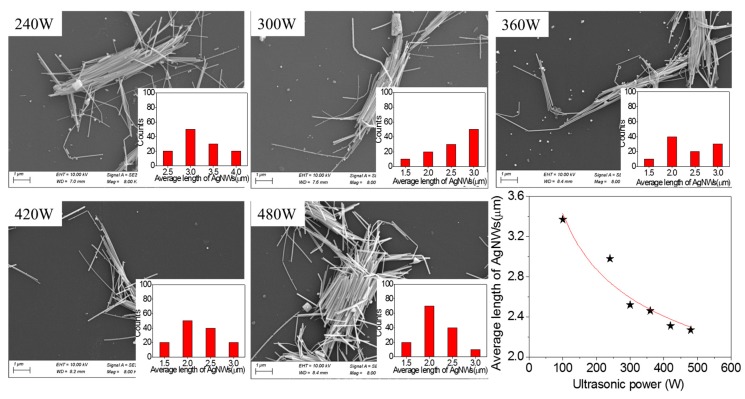
SEM images of AgNWs (1.67 mg·mL^−1^) sonicated for 3 h and different ultrasonic powers and the relationship of average length of AgNWs with ultrasonic power.

To understand more clearly the role of ultrasonic power in the length of AgNWs from sonication-induced scission, we also measured the length of AgNWs sonicated for 3 h and different ultrasonic powers, as shown in Figure 4. The inserted images are the length distributions of AgNWs. The trend of the effect of ultrasonic time on the length of AgNWs from sonication-induced scission is the same as that of Figure 2. However, the change of the length of AgNWs tends to a constant model length after ultrasonic power of 300 W. In addition, the AgNW clusters are closer. The reason may be that the AgNWs are agglomerated for long ultrasonic time. The nonlinear fitting results in a power law function relationship between the average length of AgNWs and the ultrasonic power was obtained as shown in Equation (3). Compared to Equation (2), the exponential factor has changed from −0.89 to −0.26 due to the increase in ultrasonic time. Of course, according to the previous theory, the trend between the average length of AgNWs and the ultrasonic energy is similar to that of the ultrasonic time of 1.5 h.

(3) $$L(p)\propto p^{-0.26}$$

**Figure 5:**
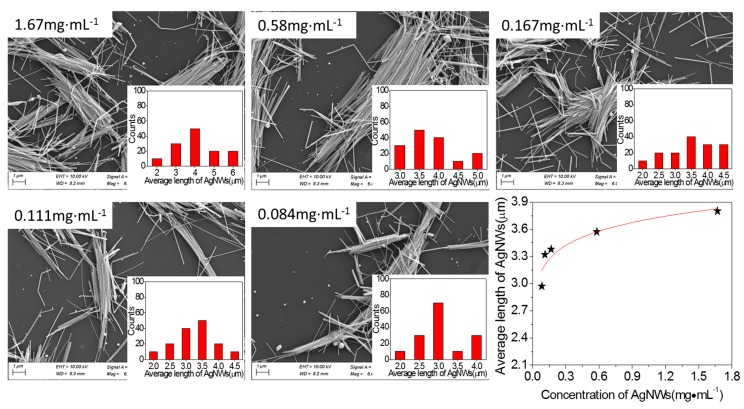
SEM images of AgNWs with different concentrations and 100 W for 3 h and relationship of the average length of AgNWs with concentration of AgNWs.

Figure 5 shows SEM images of AgNWs with different concentrations and ultrasonic power of 100 W for 3 h, and shows the relationship of the average length of AgNWs with concentration of AgNWs. The inserted images are the length distributions of AgNWs. The concentrations of AgNWs are 1.67, 0.58, 0.167, 0.111, and 0.084 mg·mL^−1^. It can be seen from Figure 5, that the length of AgNWs slightly reduces with the decrease of concentration of AgNWs. When the AgNW concentration is 1.67 mg·mL^−1^, the average length of the AgNWs is 3.98 μm, and the average length of the AgNWs decreases 0.83 μm after 20 times of dilution. Similarly, we fit the scatter data. The power law function is shown in Equation (4).

(4) $$L(c)\propto c^{0.06}$$

where *c* is the concentration of AgNWs and *L*(*c*) is the average length of AgNWs. If calculated according to the formula, the ultrasonic energy is constant under the condition that the ultrasonic time and power are constant. Therefore, this also means that the ultrasonic process relies not only on the ultrasonic energy but also on the amount of solvent in the dispersion. In other words, the average length of AgNWs is dependent on the effective ultrasonic energy acting on the acoustic cavitation.

**Figure 6:**
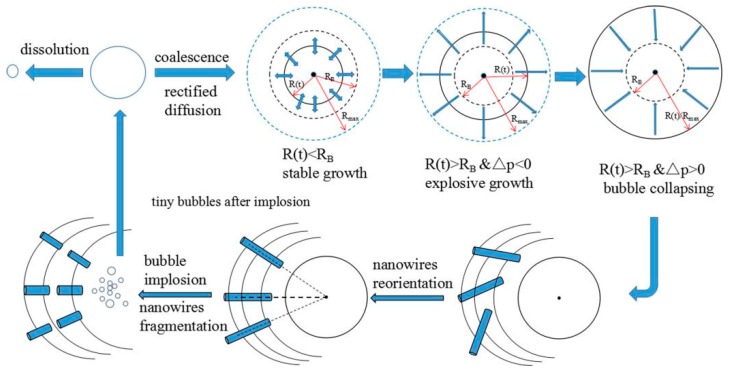
Mechanics of sonication-induced scission for fragmentation of AgNWs.

Based on the above experimental results, we present the sonication-induced scission mechanism of AgNWs as shown in Figure 6. Under the action of acoustic cavitation, small bubbles undergo three stages of nucleation, growth and explosion. When a bubble nucleates in a liquid, there are two cases: when the intensity of the sound wave is lower than a certain critical value, the bubble cores will dissolve; when the ultrasonic intensity is sufficient, the bubble grows by means of joint or rectification diffusion to reach a critical resonance [26,27,28,29,30,31,32,33,34,35,36,37]. After reaching the critical resonance size (Blake threshold), it becomes unstable, and then grows explosively. After reaching the maximum value, the small bubbles will blast instantaneously.

Under the action of the sound field, the originally disordered AgNWs are arranged in a radial order. The instantaneous blasting of the bubble produces a huge instantaneous energy, which causes AgNWs to be torn under the impact of huge energy and produces a cluster of tiny bubbles, which becomes the basis of the next bubble cycle [37,38,39,40,41,42,43,44,45]. The scission rate is governed by the supplied acoustic energy. In addition, the sonication-induced scission only affects the length of the nanowires, but not the diameter. The length of the AgNWs is proportional to the ultrasonic energy, and the longer the ultrasonic action time is, the more of the length of the AgNWs decrease linearly.

## 4. Conclusions

AgNWs of mean diameter 70 nm and mean length 12.5 μm were achieved by the polyol solvothermal method. Sonication-induced scission was used to obtain the AgNWs with different sizes. The results show that the size of AgNWs from sonication-induced scission is related to the ultrasonic time, ultrasonic power, and concentration of AgNWs. When AgNWs (1.67 mg·mL^−1^) were treated with ultrasonic power 100 W and sonicated for 6 h, the average length of AgNWs reduced to 3.31 μm. When the ultrasonic power is 240 W, the length of AgNWs (1.67 mg·mL^−1^) sonicated for 1 h reduces from 11.8 μm to 3.98 μm. When the ultrasonic power is 480 W, the average length of AgNWs (1.67 mg·mL^−1^) sonicated for 1 h reduces to 2.14 μm. When the ultrasonic power of 300–480 W were applied, the length of AgNWs linearly decreased, however, the degree of the length reduction is low. However, with extended ultrasonic time, the change of the length of AgNWs tends to a constant model length after an ultrasonic power at 300 W. The length of AgNWs slightly reduces with a decrease in the concentration of AgNWs. When silver nanowire concentration is 1.67 mg·mL^−1^, the average length of AgNWs is 3.98 μm, and the average length of AgNWs decreases 0.83 μm after 20 times of dilution. Our results show that the existence of a limiting length below which fragmentation no longer occurs.

## Figures and Tables

**Table 1:** Samples with different applied sonication energies.

Sample	1	2	3	4	5
Ultrasonic power (W)	240	300	360	420	480
Ultrasonic energy (kJ)	1296	1620	1944	2268	2592
